# Frequencies of an Immunogenic HER-2/*neu* Epitope of CD8+ T Lymphocytes Predict Favorable Clinical Outcomes in Prostate Cancer

**DOI:** 10.3390/ijms24065954

**Published:** 2023-03-22

**Authors:** Maria Goulielmaki, Savvas Stokidis, Theodoros Anagnostou, Ioannis F. Voutsas, Angelos D. Gritzapis, Constantin N. Baxevanis, Sotirios P. Fortis

**Affiliations:** 1 Cancer Immunology and Immunotherapy Center, Cancer Research Center, Saint Savas Cancer Hospital, 11522 Athens, Greece; 2 Department of Urology, Saint Savas Cancer Hospital, 11522 Athens, Greece

**Keywords:** HER-2/*neu*, prostate cancer, survival, IL-8, TGF-β, CD8+ T cell immunity, HER-2_(780–788)_

## Abstract

HER-2/*neu* is the human epidermal growth factor receptor 2, which is associated with the progression of prostate cancer (PCa). HER-2/*neu*-specific T cell immunity has been shown to predict immunologic and clinical responses in PCa patients treated with HER-2/*neu* peptide vaccines. However, its prognostic role in PCa patients receiving conventional treatment is unknown, and this was addressed in this study. The densities of CD8+ T cells specific for the HER-2/*neu*_(780–788)_ peptide in the peripheral blood of PCa patients under standard treatments were correlated with TGF-β/IL-8 levels and clinical outcomes. We demonstrated that PCa patients with high frequencies of HER-2/*neu*_(780–788)_-specific CD8+ T lymphocytes had better progression-free survival (PFS) as compared with PCa patients with low frequencies. Increased frequencies of HER-2/*neu*_(780–788)_-specific CD8+ T lymphocytes were also associated with lower levels of TGF-β and IL-8. Our data provide the first evidence of the predictive role of HER-2/*neu*-specific T cell immunity in PCa.

## 1. Introduction

T cell-mediated antitumor immunity has been recognized as a favorable biomarker for the outcome of any cancer therapy, also including immunotherapies, especially those based on immune checkpoint inhibition (ICI). It is now becoming clear that anticancer drugs mediate their beneficial therapeutic effects by generating responses de novo or reinvigorating endogenous antitumor responses [1,2]. Along similar lines, immunomodulating monoclonal antibodies reactivate pre-existing tumor-specific immunity by specifically blocking immune checkpoints or their ligands [3,4,5]. Given the importance of endogenous antitumor immune responses in the efficacy of ICI, it is conceivable that therapeutic cancer vaccines may have a beneficial role by increasing the frequencies of tumor peptide-specific T lymphocytes. 

In prostate cancer (PCa), alongside the well-known prostate-specific antigen (PSA), several reports have demonstrated the increased expression of the human epidermal growth factor receptor 2 (HER-2/*neu*) in patients with localized and more advanced hormone-resistant disease [6,7,8]. In fact, the expression of HER-2/*neu* has been found to vary among patients, mainly depending on the stage of the disease and androgen dependence, with androgen-independent, metastasized tumors being characterized by overexpression of the receptor [9,10], while patients with primary, untreated disease seem to lack identifiable HER-2/*neu* levels [11]. However, in patients with localized, previously untreated PCa, upregulation of HER-2/*neu* has been significantly associated with high Gleason scores [8,12]. Overall, HER-2/*neu* has been correlated with worse outcomes in patients with prostate cancer, including poor survival rates and an increased incidence of biochemical recurrence [13]. HER-2/*neu* has been spotlighted as a survival-promoting factor for PCa cells during the progression of the hormone-refractory disease, since it is capable of activating certain androgen receptor pathways [14,15]. Interestingly, downregulation of the HER-2 receptor has been documented in response to testosterone blockade in PCa patients [16]. Therefore, targeting HER2/*neu* represents a promising therapeutic intervention for PCa patients. 

The HER-2/*neu*_(780–788)_ nonamer has been demonstrated to induce HER2-specific human cytotoxic T lymphocytes capable of lysing HLA-A24+ human tumor cell lines, thus representing a valuable therapeutic target [17,18]. Moreover, work from our laboratory has shown that the HER-2/*neu*_(776–790)_ epitope (encompassing the HER-2/*neu*_(780–788)_ sequence) functions as a potent tumor-immunogenic epitope capable of priming CD4+ T cells to synergize with autologous CD8+ T cells for enhanced cytotoxic antitumor activity [19,20]. In our previous work [21], we reported that a peptide vaccine targeting HER-2/*neu*_(776–790)_ administered in PCa patients could expand the frequencies of CD8+ T cells specific for several HER-2/*neu* and PSA peptides, some of which are associated with clinical efficacy. However, so far, evidence for the contribution of antitumor T cell immunity to the clinical outcomes of PCa patients under standard treatments is lacking. In the present study, we showed a favorable prognostic role of the increased frequencies of CD8+ T cells specific for HER-2/*neu*_(780–788)_ in PCa patients receiving conventional therapies. 

## 2. Results

**Figure 1:**
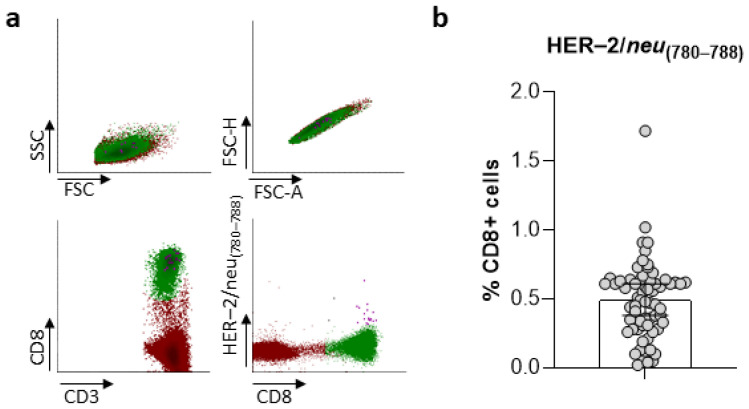
(**a**) The flow cytometry gating strategy used to analyze HLA-A*24:02-HER-2/*neu*_(780–788)_-specific CD8+ T lymphocytes identified by multiparameter flow cytometry following incubation with the MHC dextramer HLA-A*24:02+ HER-2/*neu*_(780–788)_ (PYVSRLLGI). Forward versus side scatter (FSC vs. SSC) gating was used for the identification of lymphocytes, and FSC height (FSC-H) by FSC area (FSC-A) was used for discrimination of the doublets. Then CD8+ cells were gated and the specific CD8+ cells were identified. (**b**) Densities of HLA-A*24:02-HER-2/*neu*_(780–788)_-specific T lymphocytes within the CD8+ cell subset. The results are presented as the median value with the 95% confidence interval (CI).

The frequencies of CD8+ T cells specific for HER-2/*neu*_(780–788)_ in PCa patients’ peripheral blood were determined by multiparameter flow cytometry with the MHC peptide dextramer HLA-A*24:02-HER-2/*neu*_(780–788)_. Following gating according to their FSC/SSC properties, PBMCs were subgated to CD3+ lymphocytes, which were further subgated to CD8+ T lymphocytes. HLA-A*24:02-HER-2/*neu*_(780–788)_+ lymphocytes were assessed within the gate representing CD8+ T lymphocytes after subtraction of the corresponding negative control values (Figure 1a). Following this methodology, we could detect various frequencies of CD8+ T lymphocytes specific for HER-2/*neu*_(780–788)_ among our patient cohort, which ranged from 0.02–1.72% (median = 0.49%; Figure 1b).

**Figure 2:**
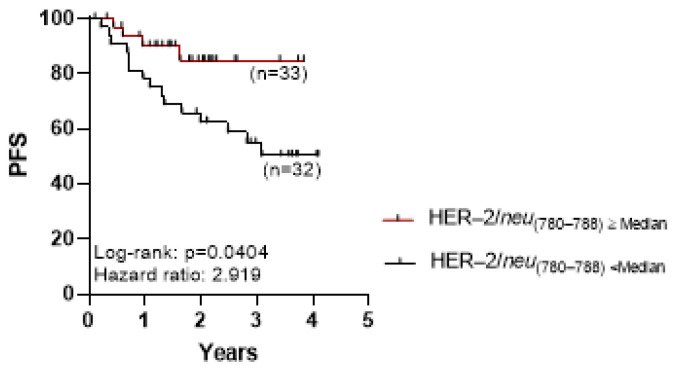
High densities of total HER-2/*neu*_(780–788)_-specific CD8+ T lymphocytes are associated with improved progression-free survival (PFS) in prostate cancer (PCa) patients. The Kaplan–Meier survival curves indicate PFS for patients with high (above or equal to the median value; red line) vs. low (below the median value; black line) frequencies of the total HER-2/*neu*_(780–788)_-specific CD8+ T lymphocytes.

Interestingly, PCa patients with high total frequencies of the HER-2/*neu*_(780–788)_-specific CD8+ T lymphocytes (above the 0.49% median frequency value and up to 1.72%; n = 33) had significantly better clinical outcomes regarding progression-free survival (PFS), compared with those with low frequencies of such CD8+ T lymphocytes (below the median value of 0.49% and down to 0.02%; n = 32) (Figure 2; *p* = 0.0404). This is an important finding, given that patients belonging to these groups did not differ significantly in their clinicopathological parameters based on age, pathologic T, PSA, and Gleason score (Table 1). The levels of HER-2/*neu*_(780–788)_-specific CD8+ T lymphocytes did not differ significantly between patients on the basis of their Gleason score (≤7, n = 31 vs. >7, n = 33; *p* = 0.4562).

**Figure 3:**
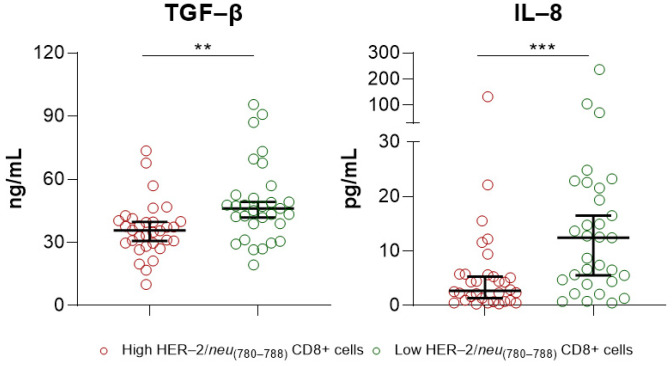
The majority of prostate cancer (PCa) patients with high frequencies of HER-2/*neu*_(780–788)_-specific CD8+ T lymphocytes had lower levels of either interleukin-8 (IL-8) or transforming growth factor beta (TGF-β). The graphs depict the association between the levels of IL-8 and TGF-β (high vs. low) in PCa patients with high or low HER-2/*neu*_(780–788)_-specific CD8+ T lymphocyte frequencies. The results are presented as median values with 95% confidence intervals (CI). ** *p* < 0.01, *** *p* < 0.001.

To obtain a better understanding of the differences in PFS, we analyzed the levels of IL-8 and TGF-β in the two PCa patient groups. Both of these play essential roles in the progression of PCa [22,23] As shown in Figure 3, the median TGF-β and IL-8 levels in patients with high frequencies of HER-2/*neu*_(780–788)_-specific CD8+ T lymphocytes were 35.73 ng/mL and 2.735 pg/mL (red circles), respectively. The median levels of both mediators were significantly higher in patients with low frequencies of HER-2/*neu*_(780–788)_-specific CD8+ T lymphocytes: 46.17 ng/mL for TGF-β and 12.44 pg/mL for IL-8 (green circles).

**Figure 4:**
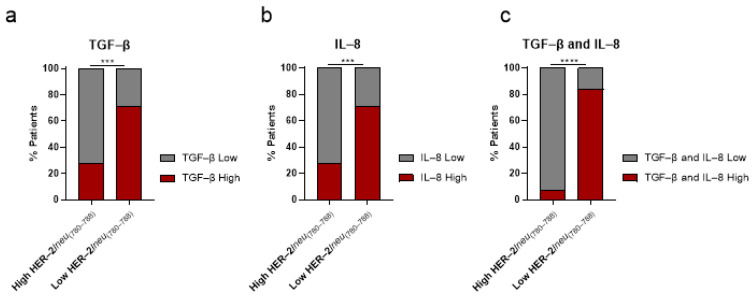
Percentages of prostate cancer (PCa) patients with high vs. low frequencies of HER-2/*neu*_(780–788)_-specific CD8+ T lymphocytes in association with high vs. low levels of (**a**) transforming growth factor beta (TGF-β), (**b**) interleukin-8 (IL-8), or (**c**) both. *** *p* < 0.001; **** *p* < 0.0001.

As also shown in Figure 4, there was a significant indirect association between the levels of IL-8 and TGF-β and the percentage of patients expressing high vs. low frequencies of HER-2/*neu*_(780–788)_-specific CD8+ T lymphocytes: 23 out of 32 PCa patients with high frequencies (72%) had lower levels of either one of these cytokines in the peripheral blood (Figure 4a,b). The multi-variable analysis that was performed to correlate the high vs. low frequencies of HER-2/*neu*_(780–788)_-specific CD8+ T lymphocytes with TGF-β or IL-8 levels revealed Pearson correlation coefficients of 0.9972 and 1.000 with p-values of 0.0473 and 0.0009, respectively. This indirect association was much more profound when TGF-β and IL-8 levels were jointly analyzed: 15 of 16 patients (93.75%) with low levels of TGF-β and IL-8 had high frequencies of HER-2/*neu*_(780–788)_-specific CD8+ T lymphocytes (Figure 4c). The opposite was true for patients with low frequencies of HER-2/*neu*_(780–788)_-specific CD8+ T lymphocytes: 22 of 31 patients (71%) had high TGF-β or IL-8 levels (Figure 4a,b) whereas 84.21% of these patients (16 of 19) had high TGF-β and IL-8 levels concomitantly (Figure 4c). The multi-variable analysis that was performed to correlate high vs. low frequencies of HER-2/*neu*_(780–788)_-specific CD8+ T lymphocytes with TGF-β and IL-8 levels (high or low) revealed Pearson correlation coefficients of 0.9991 and 0.9999, with *p*-values of 0.0138 and 0.0091 for high TGF-β and IL-8, and low TGF-β and IL-8, respectively.

## 3. Discussion

HER-2/*neu* is a tumor antigen that is found to be overexpressed in 10–30% of different types of adenocarcinomas [24]. Among its multiple tumor-promoting functions, the HER-2-dependent activation of nuclear factor-kappa B (NF-kB) relates to its enhanced invasive properties and resistance to anticancer treatments [25,26]. Moreover, proof of antitumor-adaptive suppression of immunity has been provided through plentiful mechanisms, including the persistence of immune checkpoint blockade via overexpression of PD-L1 [27] and NF-kΒ-mediated recruitment of T regulatory cells (Tregs) in the tumor microenvironment [28]. Evidently, therapeutic targeting of the receptor could potentially disrupt the imbalance between tumor dominance and antitumor immunity.

The nonamer peptide HER-2/*neu*_(780–788)_ has been reported to be immunogenic and capable of generating cytotoxic T lymphocytes recognizing and lysing HER-2/*neu*-expressing tumor cell lines [17,18]. Moreover, this peptide is encompassed in the sequence of longer helper peptides capable of stimulating tumor-reactive CD8+ T lymphocytes as well as helper antitumor responses. To this end, we [20,29] and others [30,31] have shown that the polypeptides HER-2/*neu*_(777-789)_, HER-2/*neu*_(776-788)_, and HER-2/*neu*_(776–790)_ are capable of generating, both in vivo and in vitro, specific CD4+ T lymphocytes that promote the antitumor immunity mediated by cytotoxic CD8+ T lymphocytes. Moreover, we have demonstrated that PCa patients with high T cell immunity to the native HER-2/*neu*_(776–790)_ polypeptide had better clinical outcomes when treated with a modified HER-2/*neu*_(776–790)_ polypeptide vaccine than patients with no or low pre-existing immunity [21,32]. The beneficial role of antitumor T cell immunity for the clinical outcome during or after immunotherapies has been additionally documented in patients treated with immune checkpoint inhibitors and various vaccine formulations [33,34].

In the present study, we demonstrated that PCa patients with high frequencies of HER-2/*neu*_(780–788)_-specific CD8+ T lymphocytes had better PFS compared with PCa patients with low frequencies, despite the fact that both patient groups had matched clinicopathological characteristics. We should also underline that the prognostic value of the CD8+ T cell frequencies specific for HER-2/*neu*_(780–788)_ was not influenced by the disease treatments, given that all patients included in our study were receiving various types of therapy. Ideally, our analyses should have been performed with PCa patients receiving similar treatments. However, given the small total patient cohort included in our study, stratification by the type of treatment would result in subgroups with very low numbers of patients, making statistical comparisons unattainable. 

Higher levels of HER-2/*neu* expression have been correlated with more aggressive disease, defined by a more advanced tumor stage and higher Gleason scores [35]. In addition, correlations between increased HER2/*neu* levels and a poor prognosis in prostate adenocarcinoma have been previously reported [36]. In our study, we could not detect significant differences for CD8+ T cells specifically recognizing the HER-2/*neu*_(780–788)_ epitope among PCa patients with Gleason scores less or greater than 7. Although the reasons which may account for this finding are not presently known, we could propose a link between the expression of HER-2 and the machinery of MHC Class I restricted antigen presentation, which connects the overexpression of HER-2 with downregulation of the expression of surface MHC Class I [37,38,39,40]. These defects induced by HER-2/*neu* in components of the antigen processing and presentation machinery hinder the in vivo generation of Class I restricted HER-2 derived epitopes, lowering tumor peptide recognition by CD8+ T cells [41]. Thus, higher expression of HER-2/*neu* in prostatic tumors with high Gleason scores may cause a more intense downregulation of MHC Class I antigens, accompanied by lower frequencies of HER-2/*neu* peptide-specific CD8+ T cells at similar levels to those detected in prostatic tumors with a low Gleason score. 

Importantly, we also determined a significant indirect association between the levels of IL-8 and TGF-β and pre-existing immunity. Thus, patients with high vs. low frequencies had also low or high levels of IL-8 and TGF-β, respectively. This is an important finding, given the fact that both these cytokines promote the progression of cancer via their direct and indirect actions on the master regulators of antitumor adaptive immunity, including T helper, Tregs, natural killer cells, and myeloid-derived suppressor cells [42,43,44]. Nevertheless, although we have provided strong evidence that HER-2/*neu*_(780–788)_-specific CD8+ T cell immunity is indirectly associated with the levels of IL-8 and TGF-β, additional studies will be required to determine whether these low levels of IL-8 and TGF-β are a result of the high CD8+ T lymphocyte frequencies specific for HER-2/*neu*_(780–788)_ or whether low levels of IL-8 and TGF-β allow such high CD8+ T lymphocyte frequencies to be established. These observations suggest that further investigation of the association of CD8+ T cell immunity to HER-2/*neu*_(780–788)_ with IL-8 and TGF-β levels, in which the clinical benefit in terms of evaluating the time to disease progression can be assessed in large patient cohorts, is warranted.

## 4. Materials and Methods

A review of the medical records of 65 PCa patients from the Saint Savas Cancer Hospital in Greece was performed between March 2017 and July 2022. Written informed consent was obtained from all patients enrolled. The study and the informed consent form were approved by the hospital’s IRB (IRB-ID6777/14-06-2017) and the Ethical Committee of the University of Athens (IRB-ID1516015872/03-02-2016). A tissue diagnosis of prostate adenocarcinoma was required. Patients with other primary malignancies or with a recent blood transfusion were excluded. Each patient received the appropriate treatment according to the European Association of Urology (EAU) guidelines and depending on their disease status. Patients enrolled in this prospective study had complete medical records, including baseline disease characteristics, treatments received, and clinical follow-up before and after enrolment. 

### 4.1. Blood Collection and Isolation of PBMCs 

A total volume of 20 mL blood was collected from PCa patients at the time of enrollment, which was subsequently used for HLA Class I typing and isolation of peripheral blood mononuclear cells (PBMCs). PBMCs were isolated from blood samples by Ficoll (Biochrom, Holliston, MA, USA) gradient separation at RT, washed twice with PBS, and counted in a Neubauer chamber (Poly-optik GmbH, Bad Blankenburg, Germany). Viability was always >95%. Cells were resuspended in RPMI + 40% FCS (all from Thermofisher, Waltham, MA, USA) at a concentration of 10 × 10^6^ /mL, and half of the volume of the same medium containing 20% DMSO (Applichem GmbH, Darmstadt, Germany) was added quickly at room temperature. After thorough mixing, DMSO was allowed to equilibrate through the cell membrane for 5 min before a second aliquot of the medium was added to bring the final concentration of DMSO to 10%. Next, 1 mL of the cell suspension was immediately transferred into cryovials (Thermofisher, Waltham, MA, USA) and placed in boxes at −80 °C overnight and then stored in liquid nitrogen until use. 

### 4.2. Measurements of TGF-β, IL-8, and PSA

Serum was isolated from the patients’ blood stored in BD Vacutainer™ SST™ II Advance tubes (BD, Franklin Lakes, NJ, USA), after centrifugation at 1800× *g* for 10 min at room temperature and was transferred at −20 °C until use. IL-8 (Diaclone, Besancon Cedex, France) and TGF-β (R&D Systems, Minneapolis, MN, USA) were measured in the patients’ serum by ELISA. Total PSA in the patients’ serum was measured on the fully automated chemiluminescence immunoassay (CLIA) analyzer MAGLUMI 800 (Snibe Co., Ltd., Shenzhen, China). 

### 4.3. Flow Cytometry

PBMCs were collected from the PCa patients enrolled in this study. Frozen aliquots of 10^7^ PBMCs from HLA-A*24:02+ patients were thawed in a pre-warmed RPMI 1640 culture medium supplemented with 20% FCS, 0.5 mM l-glutamine, and an antibiotic antimycotic (all from Thermofisher, Waltham, MA, USA). Cells were counted and washed twice with PBS (Thermofisher, Waltham, MA, USA). Next, the cells were stained for 20 min at room temperature in the dark with Zombie Aqua™ (Biolegend, San Diego, CA, USA) for the exclusion of dead cells. The cells were washed with PBS and 5% FCS, and then they were incubated with 10 μL of the commercially available MHC dextramer A*24:02+ HER-2/*neu*_(780–788)_ (PYVSRLLGI)-APC (Immudex, Virum, Denmark) for 20 min at room temperature in the dark, followed by staining with the specific monoclonal antibodies (all from Biolegend, San Diego, CA, USA) anti-CD14-BV510 (clone: 63D3)/anti-CD19-BV510 (clone: H1B19) for the exclusion of monocytes and B cells, respectively, and anti-CD3-PE/Cy7 (clone: UCHT1) and anti-CD8-APC/Cy7 (clone: SK1) for 20 min in the dark at room temperature. Cells were washed twice and were immediately analyzed by flow cytometry (FACSAria III, BD, Franklin Lakes, NJ, USA). For this 5 × 10^4^ CD8^+^ T cells were collected, and fluorescent minus one (FMO) samples (without the dextramer) were used as the negative controls. Data analysis was performed using Infinicyt software version 2.0.6 (Cytognos S.L., Salamanca, Spain). 

### 4.4. Statistical Analysis

GraphPad Prism v.8.0 software was used for statistical analysis of the data. Kaplan–Meier analysis with 95% confidence intervals (95% CIs) and the log-rank (Mantel–Cox) test were used for the evaluation of the association of HER-2/*neu*_(780–788)_-specific CD8+ cells with progression-free survival (PFS). The Mann–Whitney test and Fisher’s exact test were used for the statistical evaluation of patients belonging to different groups. Multi-variable analyses were performed to find correlations (Pearson correlation coefficient) between the groups of patients. High and low levels were determined as above or below the median values, respectively. Statistical differences were considered significant for *p*-values < 0.05. In the graphs, the median is presented with the 95% CI. 

## Figures and Tables

**Table 1:** Clinicopathological characteristics of the prostate cancer (PCa) patients enrolled in this study. The patients were separated in two groups based on the identified frequencies of HER-2/*neu*_(780–788)_ CD8+ cells (high vs. low).

	Patients (n = 65)			
	High HER2/*neu*_(780–788)_ CD8+ Cells (n = 33)		Low HER2/*neu*_(780–788)_ CD8+ Cells (n = 32)	
Median age; years (range)	70 (49–88) ^#^		67 (52–84) ^#^	
Median PSA; ng/mL	4.09 ^#^		3.59 ^#^	
Gleason Score > 7 *	n = 17 ^#^		n = 16 ^#^	
T status *	T1,T2 ^#^ n = 17	T3a, T3b, T4 ^#^ n = 16	T1, T2 ^#^ n = 12	T3a, T3b, T4 ^#^ n = 19

* N/A data for one patient. ^#^ *p*= n.s.

## Data Availability

The datasets were generated and analyzed during the current study with the patients’ data anonymized according to the Greek legislation for General Personal Data Protection in Research. Further information is available from the medical doctors of the study on reasonable request.

## References

[1] Zitvogel L., Galluzzi L., Smyth M.J., Kroemer G. (2013). Mechanism of action of conventional and targeted anticancer therapies: Reinstating immunosurveillance. Immunity.

[2] Aranda F., Vacchelli E., Eggermont A., Galon J., Fridman W.H., Zitvogel L., Kroemer G., Galluzzi L. (2014). Trial Watch: Immunostimulatory monoclonal antibodies in cancer therapy. Oncoimmunology.

[3] Jin C., Ali A., Iskantar A., Fotaki G., Wang H., Essand M., Karlsson-Parra A., Yu D. (2022). Intratumoral administration of pro-inflammatory allogeneic dendritic cells improved the anti-tumor response of systemic anti-CTLA-4 treatment via unleashing a T cell-dependent response. Oncoimmunology.

[4] Appleton E., Hassan J., Chan Wah Hak C., Sivamanoharan N., Wilkins A., Samson A., Ono M., Harrington K.J., Melcher A., Wennerberg E. (2021). Kickstarting Immunity in Cold Tumours: Localised Tumour Therapy Combinations With Immune Checkpoint Blockade. Front. Immunol..

[5] Ribas A., Wolchok J.D. (2018). Cancer immunotherapy using checkpoint blockade. Science.

[6] Mellinghoff I.K., Vivanco I., Kwon A., Tran C., Wongvipat J., Sawyers C.L. (2004). HER2/neu kinase-dependent modulation of androgen receptor function through effects on DNA binding and stability. Cancer Cell.

[7] Ricciardelli C., Jackson M.W., Choong C.S., Stahl J., Marshall V.R., Horsfall D.J., Tilley W.D. (2008). Elevated levels of HER-2/neu and androgen receptor in clinically localized prostate cancer identifies metastatic potential. Prostate.

[8] Shariat S.F., Bensalah K., Karam J.A., Roehrborn C.G., Gallina A., Lotan Y., Slawin K.M., Karakiewicz P.I. (2007). Preoperative plasma HER2 and epidermal growth factor receptor for staging and prognostication in patients with clinically localized prostate cancer. Clin. Cancer Res..

[9] Carles J., Lloreta J., Salido M., Font A., Suarez M., Baena V., Nogue M., Domenech M., Fabregat X. (2004). Her-2/neu expression in prostate cancer: A dynamic process?. Clin. Cancer Res..

[10] Signoretti S., Montironi R., Manola J., Altimari A., Tam C., Bubley G., Balk S., Thomas G., Kaplan I., Hlatky L. (2000). Her-2-neu expression and progression toward androgen independence in human prostate cancer. J. Natl. Cancer Inst..

[11] Okegawa T., Kinjo M., Nutahara K., Higashihara E. (2006). Pretreatment serum level of HER2/nue as a prognostic factor in metastatic prostate cancer patients about to undergo endocrine therapy. Int. J. Urol..

[12] Jorda M., Morales A., Ghorab Z., Fernandez G., Nadji M., Block N. (2002). Her2 expression in prostatic cancer: A comparison with mammary carcinoma. J. Urol..

[13] Neto A.S., Tobias-Machado M., Wroclawski M.L., Fonseca F.L., Teixeira G.K., Amarante R.D., Wroclawski E.R., Del Giglio A. (2010). Her-2/neu expression in prostate adenocarcinoma: A systematic review and meta-analysis. J. Urol..

[14] Berger R., Lin D.I., Nieto M., Sicinska E., Garraway L.A., Adams H., Signoretti S., Hahn W.C., Loda M. (2006). Androgen-dependent regulation of Her-2/neu in prostate cancer cells. Cancer Res..

[15] Shi X.B., Ma A.H., Tepper C.G., Xia L., Gregg J.P., Gandour-Edwards R., Mack P.C., Kung H.J., deVere White R.W. (2004). Molecular alterations associated with LNCaP cell progression to androgen independence. Prostate.

[16] Peixoto G.A., Korkes F., Pazeto C.L., De Castro M.G., Lima T.F.N., Wroclawski M.L., Christofe N.M., Tobias-Machado M., Santiago L.H.S., Glina S. (2021). The influence of testosterone suppression on HER2 immunoexpression in prostatic neoplastic tissue. Mol. Clin. Oncol..

[17] Shiku H., Wang L., Ikuta Y., Okugawa T., Schmitt M., Gu X., Akiyoshi K., Sunamoto J., Nakamura H. (2000). Development of a cancer vaccine: Peptides, proteins, and DNA. Cancer Chemother. Pharmacol..

[18] Ikuta Y., Okugawa T., Furugen R., Nagata Y., Takahashi Y., Wang L., Ikeda H., Watanabe M., Imai S., Shiku H. (2000). A HER2/NEU-derived peptide, a K(d)-restricted murine tumor rejection antigen, induces HER2-specific HLA-A2402-restricted CD8(+) cytotoxic T lymphocytes. Int. J. Cancer.

[19] Sotiriadou N.N., Kallinteris N.L., Gritzapis A.D., Voutsas I.F., Papamichail M., von Hofe E., Humphreys R.E., Pavlis T., Perez S.A., Baxevanis C.N. (2007). Ii-Key/HER-2/neu(776-790) hybrid peptides induce more effective immunological responses over the native peptide in lymphocyte cultures from patients with HER-2/neu+ tumors. Cancer Immunol. Immunother..

[20] Voutsas I.F., Gritzapis A.D., Mahaira L.G., Salagianni M., Hofe E.V., Kallinteris N.L., Baxevanis C.N. (2007). Induction of potent CD4+ T cell-mediated antitumor responses by a helper HER-2/neu peptide linked to the Ii-Key moiety of the invariant chain. Int. J. Cancer.

[21] Voutsas I.F., Anastasopoulou E.A., Tzonis P., Papamichail M., Perez S.A., Baxevanis C.N. (2016). Unraveling the role of preexisting immunity in prostate cancer patients vaccinated with a HER-2/neu hybrid peptide. J. Immunother. Cancer.

[22] Lopez-Bujanda Z.A., Haffner M.C., Chaimowitz M.G., Chowdhury N., Venturini N.J., Patel R.A., Obradovic A., Hansen C.S., Jackow J., Maynard J.P. (2021). Castration-mediated IL-8 promotes myeloid infiltration and prostate cancer progression. Nat. Cancer.

[23] Shackleton E.G., Ali H.Y., Khan M., Pockley G.A., McArdle S.E. (2021). Novel Combinatorial Approaches to Tackle the Immunosuppressive Microenvironment of Prostate Cancer. Cancers.

[24] Iqbal N., Iqbal N. (2014). Human Epidermal Growth Factor Receptor 2 (HER2) in Cancers: Overexpression and Therapeutic Implications. Mol. Biol. Int..

[25] Merkhofer E.C., Cogswell P., Baldwin A.S. (2010). Her2 activates NF-kappaB and induces invasion through the canonical pathway involving IKKalpha. Oncogene.

[26] Cao N., Li S., Wang Z., Ahmed K.M., Degnan M.E., Fan M., Dynlacht J.R., Li J.J. (2009). NF-kappaB-mediated HER2 overexpression in radiation-adaptive resistance. Radiat. Res..

[27] Antonangeli F., Natalini A., Garassino M.C., Sica A., Santoni A., Di Rosa F. (2020). Regulation of PD-L1 Expression by NF-kappaB in Cancer. Front. Immunol..

[28] Grinberg-Bleyer Y., Oh H., Desrichard A., Bhatt D.M., Caron R., Chan T.A., Schmid R.M., Klein U., Hayden M.S., Ghosh S. (2017). NF-kappaB c-Rel Is Crucial for the Regulatory T Cell Immune Checkpoint in Cancer. Cell.

[29] Sotiriadou R., Perez S.A., Gritzapis A.D., Sotiropoulou P.A., Echner H., Heinzel S., Mamalaki A., Pawelec G., Voelter W., Baxevanis C.N. (2001). Peptide HER2(776-788) represents a naturally processed broad MHC class II-restricted T cell epitope. Br. J. Cancer.

[30] Gillogly M.E., Kallinteris N.L., Xu M., Gulfo J.V., Humphreys R.E., Murray J.L. (2004). Ii-Key/HER-2/neu MHC class-II antigenic epitope vaccine peptide for breast cancer. Cancer Immunol. Immunother..

[31] Li Y., Ishiyama S., Matsueda S., Tsuda N., Ioannides C.G. (2008). HER-2 peptides p776 and F7, N-terminal-linked with Ii-Key tetramer (LRMK) help the proliferation of E75-TCR+ cells: The dependency of help on the side chains of LRMK-extended peptide pointed towards the T cell receptor. Oncol. Rep..

[32] Anastasopoulou E.A., Voutsas I.F., Papamichail M., Baxevanis C.N., Perez S.A. (2016). MHC class II tetramer analyses in AE37-vaccinated prostate cancer patients reveal vaccine-specific polyfunctional and long-lasting CD4(+) T-cells. Oncoimmunology.

[33] Noguchi M., Sasada T., Itoh K. (2013). Personalized peptide vaccination: A new approach for advanced cancer as therapeutic cancer vaccine. Cancer Immunol. Immunother..

[34] Sasada T., Yamada A., Noguchi M., Itoh K. (2014). Personalized peptide vaccine for treatment of advanced cancer. Curr. Med. Chem..

[35] Sanchez K.M., Sweeney C.J., Mass R., Koch M.O., Eckert G.J., Geary W.A., Baldridge L.A., Zhang S., Eble J.N., Cheng L. (2002). Evaluation of HER-2/neu expression in prostatic adenocarcinoma: A requested for a standardized, organ specific methodology. Cancer.

[36] Siampanopoulou M., Galaktidou G., Dimasis N., Gotzamani-Psarrakou A. (2013). Profiling serum HER-2/NEU in prostate cancer. Hippokratia.

[37] Lollini P.L., Nicoletti G., Landuzzi L., De Giovanni C., Rossi I., Di Carlo E., Musiani P., Muller W.J., Nanni P. (1998). Down regulation of major histocompatibility complex class I expression in mammary carcinoma of HER-2/neu transgenic mice. Int. J. Cancer.

[38] Kaplan B.L., Norell H., Callender G.G., Ohlum T., Kiessling R., Nishimura M.I. (2006). Interferon-gamma renders tumors that express low levels of Her-2/neu sensitive to cytotoxic T cells. Cancer Immunol. Immunother..

[39] Herrmann F., Lehr H.A., Drexler I., Sutter G., Hengstler J., Wollscheid U., Seliger B. (2004). HER-2/neu-mediated regulation of components of the MHC class I antigen-processing pathway. Cancer Res..

[40] Choudhury A., Charo J., Parapuram S.K., Hunt R.C., Hunt D.M., Seliger B., Kiessling R. (2004). Small interfering RNA (siRNA) inhibits the expression of the Her2/neu gene, upregulates HLA class I and induces apoptosis of Her2/neu positive tumor cell lines. Int. J. Cancer.

[41] Vertuani S., Triulzi C., Roos A.K., Charo J., Norell H., Lemonnier F., Pisa P., Seliger B., Kiessling R. (2009). HER-2/neu mediated down-regulation of MHC class I antigen processing prevents CTL-mediated tumor recognition upon DNA vaccination in HLA-A2 transgenic mice. Cancer Immunol. Immunother..

[42] Batlle E., Massague J. (2019). Transforming Growth Factor-beta Signaling in Immunity and Cancer. Immunity.

[43] Fousek K., Horn L.A., Palena C. (2021). Interleukin-8: A chemokine at the intersection of cancer plasticity, angiogenesis, and immune suppression. Pharmacol. Ther..

[44] Schalper K.A., Carleton M., Zhou M., Chen T., Feng Y., Huang S.P., Walsh A.M., Baxi V., Pandya D., Baradet T. (2020). Elevated serum interleukin-8 is associated with enhanced intratumor neutrophils and reduced clinical benefit of immune-checkpoint inhibitors. Nat. Med..

